# A Pictorial Human Case of “Furious Rabies”

**DOI:** 10.3201/eid3102.AC3102

**Published:** 2025-02

**Authors:** Antonio Perciaccante, Marina De Luca, Corinne Déchelette, Nadège Sébille, Philippe Charlier

**Affiliations:** Azienda Sanitaria Universitaria Giuliano Isontina, “San Giovanni di Dio” Hospital, Gorizia, Italy (A. Perciaccante, M. De Luca); International Society of Iconodiagnosis, Paris, France (A. Perciaccante, C. Déchelette, P. Charlier); UVSQ/Paris-Saclay, Montigny-le-Bretonneux, France (A. Perciaccante, C. Déchelette, P. Charlier); La Peau Autrement, Toulouse, France (C. Déchelette)^;^ Les Pêcheries, Musée de Fécamp, Fécamp, France (N. Sébille)

**Keywords:** Franz (Ferenc) Paczka, Un Cas Grave ou La Leçon de Médecine, A Pictorial Case of Human “Furious Rabies,” rabies, rabies virus, human furious rabies, iconodiagnosis, viruses

**Figure:**
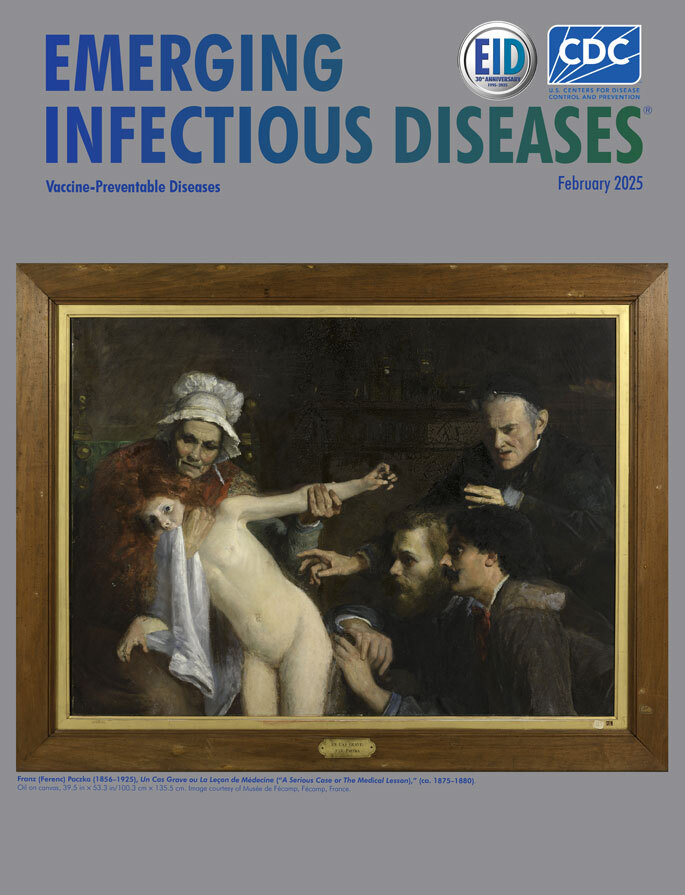
Franz (Ferenc) Paczka (1856–1925), *Un Cas Grave ou La Leçon de Médecine* (*A Serious Case or The Medical Lesson*) (ca. 1875–1880). Oil on canvas, 39.5 in × 53.3 in/100.3 cm × 135.5 cm. Image courtesy of Musée de Fécamp, Fécamp, France.

Franz (Ferenc) Paczka was a Hungarian genre painter and portraitist who studied in Paris and Rome and exhibited a few paintings at the 1878 Paris Universal Exhibition, in the Austria-Hungary section. When Paczka left France to serve in the military in his native country, he had to sell his Parisian studio collection (*1*). On April 21, 1880, an anonymous donor offered one of his paintings, considered unfinished, “*Un Cas Grave ou La Leçon de Médecine*” (*A Serious Case or The Medical Lesson*), to the fledgling Musée de Fécamp, barely 2 weeks before its official inauguration. The painting is unusual, given that Paczka usually painted portraits or small groups in calm everyday settings. Instead, it represents 2 doctors examining an unclear skin lesion on the side of a young girl who has a towel in her mouth and seems to be suffering and agitated so that she has to be held still.

There was no record as to the medical condition affecting the girl depicted in the painting. One nefarious conjecture might be that this picture may have demoniac overtones, a hypothesis that may be supported by several elements and symbols, such as the girl’s red hair, the possible presence of a priest (with his skullcap and his black clothes) behind the doctors and the way he is pointing to the “mark,” and the wound that may represent “the devil’s mark.” Moreover, in 1850–1900, France experienced a revival of occultism, spiritualism, magic, astrology, and mysticism.

However, a recently found newspaper article describing the painting and published on February 6, 1954, in Le Progrès de Fécamp by its director Jules Rolland (under the pseudonym Gihères), reveals that the lesion the doctors are examining is a dog bite wound: “‘Un cas grave’: a large canvas by Paczka. Two doctors examine suspicious spots on the body of a girl bitten by a dog. The grandfather’s and grandmother’s attitudes are fairly conventional” (*2*). This useful indication, which at that time probably appeared on the work’s label, reveals an element for an interesting retrospective diagnosis and an alternative hypothesis on this painting’s meaning.

The bite mark in a rabies victim usually heals before the onset of symptoms. However, the presence of the bite mark/scar months later might depend on the severity of the bite, whether it was subsequently infected, or the bite’s location on the body. On the basis of that information and on the evident agitation of the young girl, we speculate that this painting represents a case of the encephalic form of rabies, known as furious rabies. The painting, preserved in Musée de Fécamp, predates Émile Roux’s first inoculation of a human with rabies vaccine in July 1885; it reveals the powerlessness of families and doctors in the face of such violent symptoms and, in case of rabies, the fatal diagnosis for the victim. Cases of the disease now called rabies were recorded during the classical antiquity period (*3*). In the 4th Century BCE, Aristotle described the disease in his History of Animals*:* “Dogs suffer from three diseases … of these, rabies produces madness, and when rabies develops in all animals that the dog has bitten… it kills them; and this disease kills the dogs too” (*4*). 

Rabies is a zoonotic disease caused by viruses of the genus *Lyssavirus*, family Rhabdoviridae, order Mononegavirales. Viruses are transmitted by the saliva of the infected animal. Similar to the situation in France in the 1800s, dogs today remain the biggest concern for human rabies exposures globally, and in certain parts of the world, wildlife such as bats, foxes, jackals, mongooses, racoons, skunks, and others also transmit rabies (*5*). Rabies virus first enters in peripheral motor neurons and then passes to the central nervous system, causing signs and symptoms (*5*). Rabies can be exhibited in 2 forms—encephalitic (or furious) and paralytic—but the determining factors are not well known. Signs and symptoms depend on the form. The incubation period is 20–90 days after exposure. A short prodromal phase while the virus replicates in the dorsal root ganglia includes fever, pain, paraesthesia, pruritus, or a combination of those symptoms. In cases of furious rabies, a next acute neurologic phase is characterized by hyperactivity, confusion and agitation, hypersalivation, piloerection, hydrophobia, aerophobia resulting from electrolyte imbalance, dysphagia, hyperventilation, coordination disorders, hallucinations, and, finally, coma and death (*5*,*6*).

Primary prevention is based on animals’ vaccination to reduce the virus reservoirs. Because in most areas the primary reservoir is dogs, vaccination and elimination of stray dogs reduce virus circulation, as does dissemination of vaccine-containing bait for wild animals in rural areas. Progression to clinical disease can be prevented if wound care and postexposure prophylaxis by immunoglobulin and vaccination are administered rapidly after animal bite, thus sparing many people around the world from suffering the same fate as the young girl depicted by Paczka in his painting. Unfortunately, the therapeutic is not available everywhere.

Although Louis Pasteur developed the first effective rabies vaccines for humans in the 19th Century, today, the virus is still endemic among animals in some regions of the world (i.e., throughout Asia and Africa), and human rabies remains a serious public health challenge (*6*,*7*). *Un Cas Grave ou La Leçon de Médecine* is an example of how art can tell the history of medicine and focus on a health issue that is perhaps underestimated today.
